# Rank-Two NMF Clustering for Glioblastoma Characterization

**DOI:** 10.1155/2018/1048164

**Published:** 2018-10-23

**Authors:** Aymen Bougacha, Ines Njeh, Jihene Boughariou, Omar Kammoun, Kheireddine Ben Mahfoudh, Mariem Dammak, Chokri Mhiri, Ahmed Ben Hamida

**Affiliations:** ^1^ATMS-ENIS, Advanced Technologies for Medicine and Signals, Department of Electrical and Computer Engineering, National Engineers School, Sfax University, Sfax, Tunisia; ^2^Department of Radiology, University Hospital Habib Bourguiba, Sfax, Tunisia; ^3^Department of Neurology, University Hospital Habib Bourguiba, Sfax, Tunisia

## Abstract

This study investigates a novel classification method for 3D multimodal MRI glioblastomas tumor characterization. We formulate our segmentation problem as a linear mixture model (LMM). Thus, we provide a nonnegative matrix *M* from every MRI slice in every segmentation process' step. This matrix will be used as an input for the first segmentation process to extract the edema region from T2 and FLAIR modalities. After that, in the rest of segmentation processes, we extract the edema region from T1c modality, generate the matrix *M*, and segment the necrosis, the enhanced tumor, and the nonenhanced tumor regions. In the segmentation process, we apply a rank-two NMF clustering. We have executed our tumor characterization method on BraTS 2015 challenge dataset. Quantitative and qualitative evaluations over the publicly training and testing dataset from the MICCAI 2015 multimodal brain segmentation challenge (BraTS 2015) attested that the proposed algorithm could yield a competitive performance for brain glioblastomas characterization (necrosis, tumor core, and edema) among several competing methods.

## 1. Introduction

Brain tumor represents 85% to 90% of all primary central nervous system tumors. It is one of the main sources for the increase in death rate among children and adults in the world. Bauer et al. [1] noted that glioma could be considered as the largest common brain tumor with the maximum death rate. According to its severity, such brain tumor could be classified as low-grade glioblastomas (LGG) and high-grade glioblastomas (HGG). The low-grade tumors keep developing for many years and could be designed as slow invaders of brain safety tissue. On the other hand, the high-grade tumors known as glioblastomas multiform (GBM) are incurable with an average life of one year after its revelation. Such invasive tumors are very heterogeneous due to their morphological, cytological, and molecular variability. It might have a variety of shapes, might be of any size, and might appear at any location and in different image intensities.

In behalf on their frequency and severity, glioblastomas continue to be the major therapeutic issue for neurosurgeons, neuro-oncologists, and radiation therapists.

The magnetic resonance imaging (MRI) could be considered as one of the main noninvasive modalities used to explore glioblastomas brain tumor for diagnosis, evaluation as well as for inspection of the addressed treatment effect. Such procedure offers the generation of different sequences by modifying the excitation and the repetition times during the acquisition of the image. Each sequence provides relevant structural information. The main four standard MRI modalities are the T1-weighted MRI (T1), the T2-weighted MRI (T2), the T1-weighted MRI with gadolinium contrast enhancement (T1-Gd), and the fluid-attenuated inversion recovery (FLAIR). Conventionally, T1 images are used to differentiate healthy tissue, while T2 images provide a light signal on the image which helps to delineate the region of the edema. In T1-Gd images, the hyperintense given by the accumulated contrast agent (gadolinium ions) in the active cellular region of the tumor tissue allows us to facilitate the observation of the tumor boundary. The necrotic cells are observed by a hypointense part of the tumor core, as they do not interact with the contrast agent, which makes them easily distinguishable from the active cell region. In FLAIR images, the suppression of the signal of molecule water provides a good observation of edema region from cerebrospinal fluid (CSF).

Glioblastomas segmentation is a challenging task that could be considered as an essential preprocessing task in brain tumors diagnoses. Manual segmentation is a tedious and a time-consuming process for radiologists.

Consequently, automatic segmentation algorithms would be recommended in order to obtain accurate and reliable brain tumor delimitation but it remains a persistent challenge due to the structural complexity of glioblastomas tumors. Furthermore, such tumors present essentially four different zones: edema, which represents an excess accumulation of fluid in the intracellular or extracellular spaces of the brain, nonenhancing solid core, necrotic/cystic core, and enhancing core.

Several research studies have been investigated to segment different tumor zones in multiple MRI modalities (T1, T1-Gd, T2, and Flair) [1–3]. Dupont et al. [4] present, in their review, four main classes in order to segment glioblastomas tumor: region-based approach, edge-based approach, and classification-based algorithms approach.

In the region-based approach, we intend to implement segmentation by merging neighbourhood pixels that have similar characteristics. A region-based method presented by Franz et al. [5] is used to differentiate the enhanced tumor portion, the necrotic zone, and the edema zone. Only two modalities (T1-Gd and Flair) have been used as an input for this algorithm, and only the image intensity has been employed as a feature in order to delimit different tumor region's zone. As a consequence, coherent intensity pixels have been grouped into three classes: tumor enhancement zone, necrosis zone, and edema zone. Sachdeva et al. [6] introduced an edge-based method based on image texture's intensity and a specific active contour to achieve semiautomatic segmentation. The authors used multimodal MRI (T1-weighted, T2-weighted, and T1-Gd MRI) to test their algorithm. Essidike et al. [7] proposed a two-step brain tumor segmentation. For the first step, a numerical simulation of the optical correlation has been used to detect brain tumor, and an active contour model is used to detect region for the next step.

Healthy tissues extraction can help to provide GBM structure segmentation, and atlas-based approaches have been used in this way. Prastawa et al. [8] introduced an automatic brain tumor segmentation with edema's detection. This algorithm used only T2 MRI. Pixels classification of cerebrospinal fluid (CSF), white matter (WM), and gray matter (GM) was performed from atlas template. The unclassified pixels have been labelled as tumor or edema.

Classification approaches are widely used in image segmentation. These methods consist in clustering pixels depending on different features used as an input vector (intensity, texture, neighbours, and spatial distribution in the image) of a clustering algorithm. There are two groups of classification approaches: supervised approaches or unsupervised approaches. Wu et al. [9] applied a supervised method. A multimodal MRI is segmented into superpixels to minimize the sampling problem. Then, features were extracted from the superpixels using multilevel Gabor wavelet filters. These features are used to power the support vector machine (SVM) model. The theory of conditional random fields has been applied to segment the tumor based on the output of the SVM models. Finally, the marking noise was removed using “structural knowledge.” This system was applied with 20 GBM cases. Recent studies [10–12] have used the deep learning technique for the segmentation of GBM tumors. These methods of segmentation differ according to the training concept. Havaei et al. [10] performed a modified conventional neural network (CNN) and a two-phase training to touch on problems related to the unbalance of GBM labels. Zhao et al. [12] used a three-segmentation model based on fully convolutional neural networks (FCNNs), conditional random fields (CRFs), and recurrent neural networks (RNNs). These models are trained with 2D image patches and slices acquired in axial, coronal, and sagittal views, respectively, and mixed them to segment brain tumors. Hussain et al. [11] implemented a deep conventional neural network (DCNN) where two networks are piled over one another to construct a new linear nexus architecture. The first network holds in parallel placing of layers, whereas in the second network, layers are structured linearly.

Corso et al. [13] proposed a Bayesian model classification. This unsupervised method has used two concepts: class model and graph cuts. The objective was to fuse speed of graph cuts and statistical distribution efficiency of the class model. The proposed method was executed to twenty GBM cases with T1, T1-Gd, T2, and FLAIR previously investigated by experts. Presented as one of the popular unsupervised clustering methods, Cordova et al. [14] developed a fuzzy c-means GBM segmentation using T1-GD images. This method has been tested with thirty seven cases. In [15, 16], authors applied a hierarchical nonnegative matrix factorization (hNMF) on multiparametric MRI to provide tissue characterization. The specification of tissue's patterns was obtained, and the spatial distribution of each tissue type was visualized. Li et al. [17] also applied hNMF to brain MRSI data for GBM tissue's differentiation.

**Figure 1:**
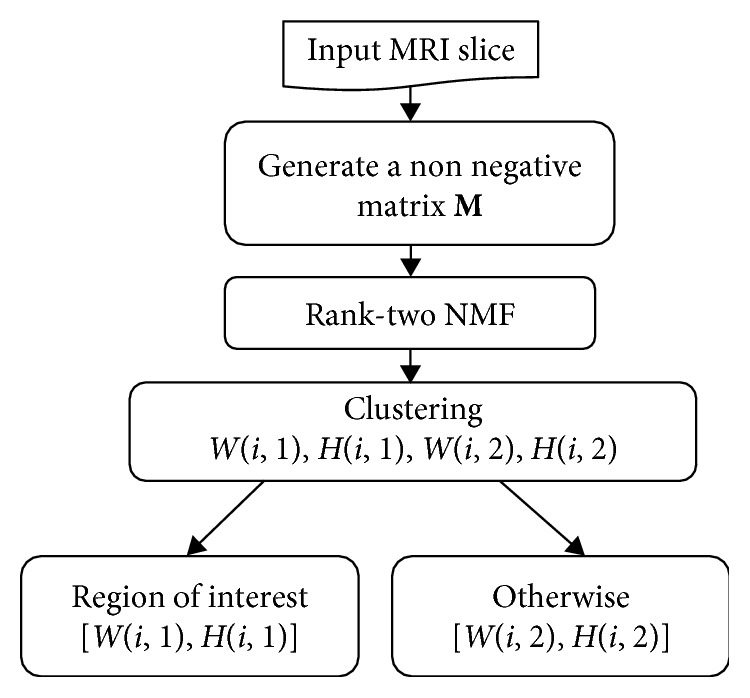
Segmentation process.

In this work, we propose a novel classification method for 3D multimodal MRI glioblastomas tumor characterization. We formulate our segmentation problem as a linear mixture model (LMM). Thus, we provide a nonnegative matrix *M* from every MRI slice in every segmentation process' step. This matrix will be used as an input for the first segmentation process to extract edema region from T2 and FLAIR modalities. After that, in the rest of segmentation processes, we extract the edema region from T1c modality, generate the matrix *M* and segment the necrosis, the enhanced tumor, and the nonenhanced tumor regions as described in the method's flowchart (see Figure 1). In the segmentation process, we apply a rank-two NMF clustering which could be defined as a blind source separation technique [18]. It consists in approximately the factorization of a matrix *M* into the product of a source matrix *W* and an abundance matrix *H*. This method has been used as a brain tumor segmentation with MRSI (magnetic resonance spectroscopy image) [17] and multiparametric MRI data [15, 16]. The main contribution of this study and the differences between our work and others mentioned previously lies on the application of the GLCM features for nonnegative matrix *M* and the use of a rank-two NMF instead of the hierarchical NMF. The proposed method does not require a training dataset, as is the case of the many existing methods. Quantitative assessment over the publicly existing training and testing dataset from the MICCAI Multimodal Brain Tumor Segmentation 2015 (BraTS 2015) challenge [19] confirm that the proposed method provides a competitive performance.

The remainder of this paper is arranged as follows: the materials and methods section where we define the Multimodal Brain Tumor Segmentation Benchmark (BraTS 2015) data and illustrate the segmentation methodology. The results and discussion shows the experimental results with a discussion. Finally, the conclusion section illustrates various perspectives of this work.

## 2. Materials and Methods

**Figure 2:**
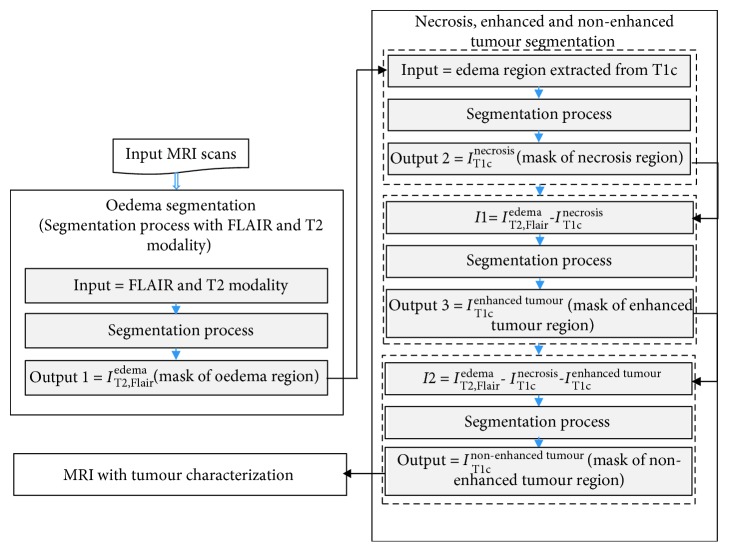
Flowchart of the proposed GBM characterization.

The obtained results were based on approved evaluations using the Multimodal Brain Tumor Segmentation Benchmark (BraTS 2015) [19]. In this section, we present in details the used dataset, the evaluation metrics, and the different steps of the proposed methodology: the preprocessing step, the feature extraction, and the rank-two NMF segmentation. The proposed approach could be outlined according to the flowchart (see Figure 2).

### 2.1. Multimodal Brain Tumor Segmentation Benchmark (BraTS 2015)

The Multimodal Brain Tumor Segmentation dataset (BraTs 2015) is in continuation of BraTS 2012, BraTS 2013, and BraTS 2014. It has been organized by B. Menze, M. Reyes, K. Farahani, and J. Kalpathy-Cramer in conjunction with the MICCAI 2015 conference. This available publicly training and testing dataset could be considered as very useful to compare the existing method and to gauge the current state of the art in brain tumor segmentation. It consists in comparing and evaluating 3D MRI brain tumor regions obtained by segmenting multimodal imaging dataset. Such task could be considered as a challenging task in medical image analysis due to the unpredictable appearance and shape of glioblastomas tumor. The coregistered, the skull-stripped, and the annotated training dataset are available via the Virtual Skeleton Database (VSD) [20].

Training dataset, testing dataset, and the ground truth are stored as signed 16-bit integers, but only positive values are used. Four MRI modalities are proposed for each case: T1 modality, T2 modality, T1c modality, and FLAIR modality. The manual segmentations (ground truth) of the patient images have the following five different labels: (1) for necrosis, (2) for edema, (3) for nonenhancing tumor, (4) for enhancing tumor, and (0) for everything else.

The evaluation is done for 3 different tumor subcompartments:

- *Region* 1. Complete tumor (labels 1 + 2 + 3 + 4 for patient data and labels 1 + 2 for synthetic data)
- *Region* 2. Tumor core (labels 1 + 3 + 4 for patient data and label 2 for synthetic data)
- *Region* 3. Enhancing tumor (label 4 for patient data and n.a. for synthetic data)

The total case of training data is 274 patients (220 high-grade tumors and 54 low-grade tumors), while the testing dataset contains 110 subjects with low-grade glioma (LGG) and high-grade glioma (HGG).

### 2.2. Evaluation Metric

In this study, the dice (DM) [21] and the sensitivity metrics were used to evaluate the quantitative performance and the quality of segmentation. This requires computing the similarities between ground-truth segmentations provided with BraTS 2015 dataset and the obtained results. These metrics take values within the interval [0...1], where 1 indicates a perfect match and 0 a complete mismatch. An automated segmentation should be uploaded directly to the evaluation page to obtain the dice metric score [20].

### 2.3. Proposed Algorithm

In this work, we present a performed algorithm in order to segment the different tumor regions (necrosis, edema, nonenhancing tumor, enhancing tumor, and everything else). The proposed methodology is tested and validated using BraTS 2015. We present the flowchart that describes the segmentation process (see Figure 1).

**Figure 3:**
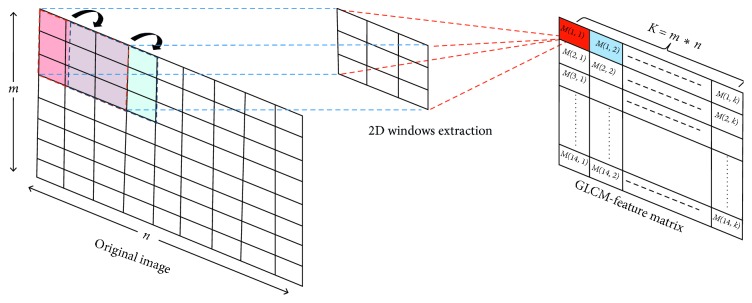
GLCM-feature matrix *m* generation.

We formulate our segmentation problem as a linear mixture model (LMM). As depicted in Figure 1, from an input MRI scan, we generate a nonnegative matrix *M*, that is, matrix *M* ∈ ℝ_+_^*m∗n*^ with *m* features and *n* voxels as follows (see Figure 3): the (*i*,*j*)th entry *M*(*i*; *j*) of the matrix *M* is the ith GLCM feature of the *j*th voxel. Hence, each column of *M* is equal to the feature signature of a voxel while each row is a vectorized image at a given feature. The linear mixing model (LMM) assumes that the feature signature of each voxel is a linear combination of the feature signatures of the constitutive pattern in image (endmembers), where the weights in the linear combination are the abundances of each endmember in this voxel [22].

Supposing the image encloses *r* endmembers, and designating *W*(:,*k*) ∈ ℝ^*m*^(1 ≤ *k* ≤ *r*) the feature signatures of the endmembers, we can write the LMM as

(1) $$\begin{array}{c} M\left(:,j\right)=\overset{r}{\underset{k=1}{\sum }}W\left(:,k\right)H\left(k,j\right),\;1\leq j\leq n, \end{array}$$

where *H*(*k*, *j*) is the abundance of the kth endmember in the jth voxel, so ∑_*k*=1_^*r*^*H*(*k*, *j*)=1 for all *j*, which is specified to as the abundance sum-to-one constraint. As all matrices concerned *M*, *W* and *H* are nonnegative; the LMM is corresponding to nonnegative matrix factorization (NMF). Having a nonnegative matrix *M* ∈ ℝ_+_^*m∗n*^ and a factorization rank *r*, discover two nonnegative matrices *W* ∈ ℝ_+_^*m∗r*^ and *H* ∈ ℝ_+_^*r∗n*^ such that *M* ≈ *WH*. However, having an MRI slice with *r* endmembers, it consists in to cluster the pixels into *r* clusters, and each cluster is equivalent to one endmember. Mathematically, having a matrix *M* ∈ ℝ_+_^*m∗n*^, we aim to find *r* disjoint clusters *C*_*k*_ ⊂ {1, 2,…, *n*} for 1 ≤ *k* ≤ *r* so that ∪_*k*=1,2,…,*r*_*C*_*k*_={1, 2,…, *n*} and so that all pixels in *C*_*k*_ are monopolized by the same endmember.

**Figure 4:**
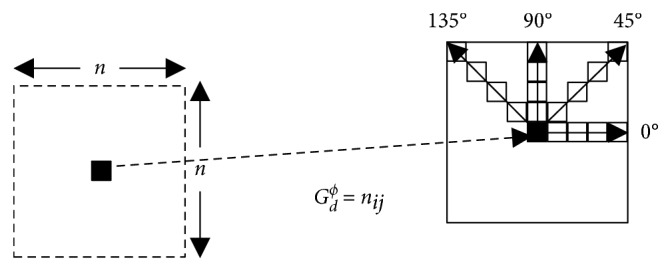
2D GLCM computation for *n* *∗* *n* window. Main directions (0°, 45°, 90°, and 135°) and a distance *d* are used. Mean value is affected to central voxel.

MRI imaging systems provide images with high resolution and high tissue contrast. These images are defined with a depth of up to 16 bits corresponding to 65535 intensity levels. In order to simplify the calculation, all intensity values were rearranged to a gray level values with a maximum of 255, and texture features are computed using the grayscale co-occurrence matrix (GLCM). GLCM has been useful in various image processing fields. It is a squared matrix G(*N*,*N*) where *N* represents the number of gray level existing in the window. This matrix is a structure that represents the co-occurring intensity values at a given offset. This is defined by the fact that the GLCM gives information on how often a gray level arise at different directions and a distance *d*. Usually, four directions are looked up in the 2D case: *ϕ* = 0°, *ϕ* = 45°, *ϕ* = 90°, and *ϕ* = 135°. The structure of the 2D GLCM is shown in Figure 4, where *n*_*ij*_ is the number of co-occurrences of gray levels *i* and *j* at a specific direction *ϕ* and a distance *d*. Haralick et al. [23] defined texture features calculated using the GLCM. Moreover, Haralick recommended utilizing the average value of the features calculated for the four directions to ensure rotation invariance.

The GLCM features (Table 1) used in this study were extracted at a distance *d* = 1 with mean value for four directions.

As illustrated in Figure 2 the MRI modalities are used as follows: from T2 and Flair modalities, we apply the segmentation process in order to obtain the edema mask region *I*_T2,Flair_^edema^. Then, we apply the obtained mask on T1_C_ modality and we apply the segmentation process in order to obtain the necrosis region's mask *I*_T1c_^necrosis^. We calculate, after that, the intermediary image I1 = *I*_T2,Flair_^edema^-*I*_T1c_^necrosis^ which will be used as an input to the segmentation process in order to obtain the enhanced tumor region‘s mask *I*_T1c_^enhanced  tumour^. We also calculate a second intermediary image I2 = *I*_T2,Flair_^edema^-*I*_T1c_^necrosis^-*I*_T1c_^enhanced  tumour^, and we apply the segmentation process in order to obtain the nonenhanced tumor region's mask *I*_T1c_^nonenhanced  tumour^.

As we can see in Figure 2 that in every segmentation process, we aim to cluster the input MRI slice into two clusters. However, we propose to use a rank-two NMF clustering. Having a nonnegative matrix *M* ∈ ℝ_+_^*m∗n*^, rank-two NMF searches two nonnegative matrices *W* ∈ ℝ_+_^*m∗*2^ and *H* ∈ ℝ_+_^2*∗n*^, where *M* ≈ *WH*. This factorization is a two-dimensional description of the data; more literally, it conceives the columns of *M* onto a two-dimensional pointed cone developed by the columns of *W*. Therefore, the approach to segment the MRI slice, in other words to cluster the columns of *M*, is to selecting the clusters like this: *C*_1_={*i*|*H*(*i*, 1) ≥ *H*(*i*, 2)}, which represents the region of interest and *C*_2_={*i*|*H*(*i*, 1) < *H*(*i*, 2)} represents the otherwise zone.

## 3. Results and Discussion

In this section, we report the segmentation result obtained by the proposed method over the publicly training dataset from BraTS 2015. We also present the quantitative evaluation by computing the dice metric and the sensitivity for, respectively, complete tumor, tumor core, and the enhancing tumor. This section is supported by illustration that depict typical example of the obtained results.

**Figure 5:**
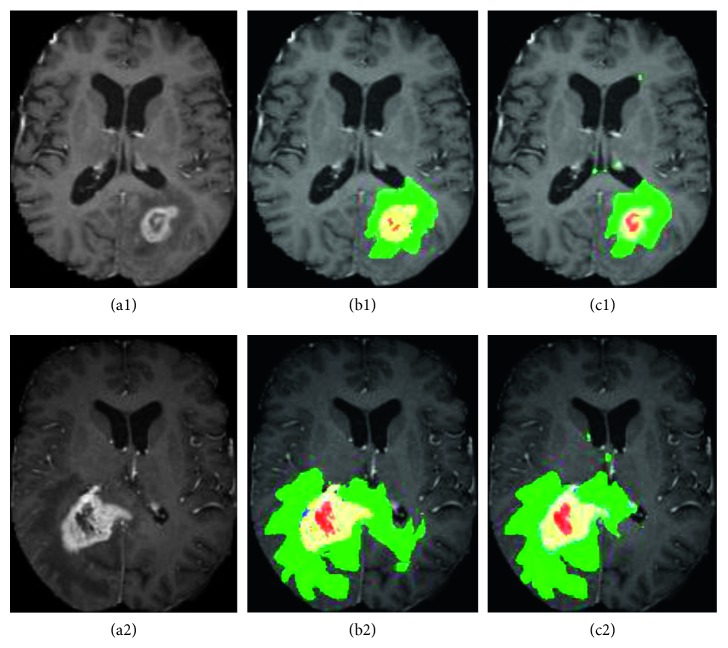
Examples of characterization results obtained from rank-two NMF methods on BRATS 2015 data. T1c images with high-grade tumor case HG-02 (a1), HG-03 (a2); b1-b2 ground truth; c1-c2 results using rank-two NMF; edema (green), necrosis (red), enhanced tumor (yellow), and nonenhanced tumor (blue).

Figure 5 depicts the segmentation obtained on two high-grade gliomas from the training dataset. The green zone corresponds to the edema region, the yellow zone represents the enhanced tumor, the red zone is the necrosis, and the blue color represents the nonenhanced tumor.  Table 2 attests the performance of the proposed algorithm by a greet score for dice and sensitivity metric.

The segmentation methodology proposed in this paper can process an immense diversity of tumors because it does not depend on contrast enhancement. It segments the whole brain, including healthy tissue types, and automatically identifies edema, nonenhanced, enhanced tumor, and necrosis region. Delineating the edema region can be valuable for surgical planning and description of radiation therapy fields, and since the edema region demonstrates the volume over which the tumor applies obvious chemical effects, recognition of areas of interest to various investigators is involved in tumor growth and treatment. Delineating the edema region can also be valuable for surgical planning and radiation therapy. Often, edema regions need to be treated to minimize the risk of recurrence.

We have carried out the proposed method to MR data from patients with glioblastoma tumors. These images include tumors with different intensities, sizes, locations, and shapes. This authorizes us to demonstrate the large field of application of our algorithm.

We have executed our tumor characterization method on BRATS 2015 challenge dataset. Two cases have been selected randomly in this experiment. Definitely, there are four label types in this dataset, including necrosis, edema enhanced, and nonenhanced tumor. As pointed out in Table 2, the dice ratio is superior to 0.85, illustrating good overlap with ground truth. Moreover, the sensitivity is superior to 0.8 which means that the segmentation results are reliable enough.

The results of this table also illustrate that the quality of the segmentation for whole tumor is better than for core tumors because of their well-defined boundaries. Enhancement of the approach for segmenting core tumors could still be valuable.

## 4. Conclusion

In this paper, we define a novel methodology for 3D multimodal MRI GBM tumor characterization. Unlike from the classic tumor segmentation methods, in the proposed method, we observe the brain tumor segmentation task as a four-class (tumor (including necrosis, enhanced, and nonenhanced tumor), edema, and normal tissue) classification problem regarding three modalities T2, FLAIR, and T1c. We formulate our segmentation problem as a linear mixture model (LMM). Thus, we provide a nonnegative matrix *M* from every MRI slice in every step of the segmentation process. This matrix will be used as an input for the first segmentation process to extract edema from T2 and FLAIR modality. After that, in the rest of segmentation processes, we extract the edema region from T1c modality, generate the matrix *M* from this modality, and segment necrosis, enhanced tumor, and nonenhanced tumor regions. In the segmentation process, we apply a rank-two NMF clustering. Compared to the traditional tumor segmentation methodologies, the proposed method is easy to achieve and quite robust to high-intensity inhomogeneity images. Comparison results on BRATS 2015 challenge dataset illustrate the superior achievements of the proposed method.

As a perspective, we will apply the proposed method through all training data and also the proposed testing data in order to attest the performance of the algorithm.

## Figures and Tables

**Table 1:** GLCM features.

Feature	Formula	Feature	Formula
Contrast	∑_*p*=1_^*N*^(*p*^2^∑_*i*=1_^*N*^∑_*j*=1_^*N*^*G*_*d*_^*ϕ*^(*i*, *j*)), *p*=|*i* − *j*|	Sum average	∑_*i*=1_^2*N*−1^(*ip*_*x*+*y*_(*i*))
Energy	∑_*i*=1_^*N*^∑_*j*=1_^*N*^*G*_*d*_^*ϕ*^(*i*, *j*)^2^	Cluster shade	∑_*i*=1_^*N*^∑_*j*=1_^*N*^(*i*+*j* − *μ*_*x*_ − *μ*_*y*_)^3^*G*_*d*_^*ϕ*^(*i*, *j*)
Dissimilarity	∑_*i*=1_^*N*^∑_*j*=1_^*N*^(*i* − *j*)*G*_*d*_^*ϕ*^(*i*, *j*)	Cluster prominence	∑_*i*=1_^*N*^∑_*j*=1_^*N*^(*i*+*j* − *μ*_*x*_ − *μ*_*y*_)^4^*G*_*d*_^*ϕ*^(*i*, *j*)
Entropy	−∑_*i*=1_^*N*^∑_*j*=1_^*N*^*G*_*d*_^*ϕ*^(*i*, *j*)log(*G*_*d*_^*ϕ*^(*i*, *j*)	Maximum probability	∑_*i*=1_^*N*^∑_*j*=1_^*N*^max_*i*,*j*_{*G*_*d*_^*ϕ*^(*i*, *j*)}
Correlation	$\sum_{i=1}^{N}\sum_{j=1}^{N}G_{d}^{\phi }\left(i,j\right)\frac{\left(i-\mu_{x}\right)\left(i-\mu_{y}\right)}{\sigma_{x}\sigma_{y}}$	Difference variance	∑_*i*=1_^2*N*−1^(*i*^2^*p*_*x*−*y*_(*i*))
	*μ*_*x*_=∑_*i*=1_^*N*^∑_*j*=1_^*N*^*jG*_*d*_^*ϕ*^(*i*, *j*), *μ*_*y*_=∑_*i*=1_^*N*^∑_*j*=1_^*N*^*iG*_*d*_^*ϕ*^(*i*, *j*)		
	*σ*_*x*_=∑_*i*=1_^*N*^∑_*j*=1_^*N*^(*j* − *μ*_*x*_)^2^*G*_*d*_^*ϕ*^(*i*, *j*)		
	*σ*_*y*_=∑_*i*=1_^*N*^∑_*j*=1_^*N*^(*i* − *μ*_*y*_)^2^*G*_*d*_^*ϕ*^(*i*, *j*)		
Homogeneity	$\sum_{i=1}^{N}\sum_{j=1}^{N}\frac{1}{1+{\left(i-j\right)}^{2}}G_{d}^{\phi }\left(i,j\right)$	Autocorrelation	∑_*i*=1_^*N*^∑_*j*=1_^*N*^(*i∗j*)*G*_*d*_^*ϕ*^(*i*, *j*)
Variance	∑_*i*=1_^*N*^∑_*j*=1_^*N*^(1 − *μ*)^2^*G*_*d*_^*ϕ*^(*i*, *j*)	Sum entropy	−∑_*i*=1_^2*N*−1^(*p*_*x*+*y*_(*i*)log(*p*_*x*+*y*_(*i*)))
Difference entropy	−∑_*i*=0_^*N*−1^(*p*_*x*−*y*_(*i*)log(*p*_*x*−*y*_(*i*)))	Sum variance	−∑_*i*=1_^2*N*−1^(1 − SumEnt)^2^*p*_*x*+*y*_(*i*)

**Table 2:** Quantitative results for the BRATS 2015 MRI images.

Class	Dice	Sensitivity
Complete tumor	0.87	0.84
Tumor core	0.77	0.64
Enhancing tumor	0.74	0.61

## Data Availability

The BRATS 2015 data used to support the findings of this study are included within the article.
